# Recuperation

**Published:** 1892-03-26

**Authors:** 


### The Doctor's Armchair.

RECUPERATION.

"To most of Lord Hampden's friends," says the *Daily Graphic*, "the news of his death must have been a surprise. Until recently he was the embodiment of rude health. Lithe in form, ruddy in countenance, he carried his seventy-eight years like a Gladstone, and few would have imagined on seeing him strolling through a Sussex lane that for twelve years of the severest strife and turmoil he had filled the presidential chair at St. Stephen's. In the cheery face, the bright eye, and brisk walk, there was no trace of the all-night sittings or the exciting scenes in which the late Speaker played so onerous a part. Such was the recuperative effect of country life."

Recuperation is the very thing of all others which men who work, and women who wear themselves out with family cares, are constantly wishing for, but seldom know where to find. The labour of life is a strenuous business; much more strenuous than most of us perceive it to be. A man feels tired, a woman feels weary and exhausted, without reflecting that it might be desirable to enquire into the physiology of fatigue and exhaustion. But just as a knowledge of railway travelling diminishes the toil and worry of a long journey, and as a knowledge of the art of war conserves human life and conduces to success in battle, so a rational understanding of the capacities, the powers, and the limitations of the human body and mind tends to the diminution of wear and tear, to enjoyment, and to long life.

Mr. Matthew Arnold was insistent in his accusations of what he called "Philistinism" against the British public. By "Philistinism," Mr. Arnold gives us to understand that he chiefly means "inaccessibility to ideas." Sometimes he is quite pathetic on this point, but still oftener he is stinging and sharp. It can hardly be denied that the armour of self-approval which surrounds a great many fairly educated Britons is of triple brass. They are most decidedly "inaccessible to ideas." The philosophic physician and physiologist, of which class there are not a few in this country at the present time, tries very hard indeed to get the invaluable knowledge which his experience has furnished him with, translated into the current mental coin of his period, and converted into fact in the daily experience of his contemporaries. But the task is prodigious. It is surprising to see clever men of business, and still more men of the pen, whose daily work it is to convey instruction to the public, positively refusing to take any trouble whatever to acquire the most elementary scientific knowledge of the powers, capacities, and limitations of their own bodies and minds. Not only so, but those very people, who regard a physiological idea as our grandfathers used to regard a Frenchman, with positive hatred, will yet boldly accuse the members of the medical profession of ignorance and incapacity. From a physiological stand-point, the medical man cannot but have a good deal of sympathy with Mr. Matthew Arnold in the charge of Philistinism which he brings against the average Briton.

The question of recuperation is one of which the importance is not accidental or insignificant, but quite fundamental. We must recuperate or we shall wear out and die prematurely. "It is better to wear out than rust out," says one. Of course, but it is best of all to wear out only at the proper rate of speed, and to use our energies in such a way that they shall produce the largest and worthiest results for our families, the world, and ourselves. Recuperation is not, after all, so very unfamiliar an idea. If a man's watch begins to drag in recording time, he recuperates it at the watchmaker's, by cleaning and oiling it. If his hunter gets soon weary, and fails at a very low fence, he gives him a week or ten days' rest, with gentle exercise, and a little alterative and tonic medicine. Many hard-working men, indeed, admit the necessity of recuperating themselves; but they carry out the thing unintelligently, unsystematically, and only at long intervals. Recuperation, to do all which it is capable of doing, must be constant as well as occasional, and occasional as well as constant.

Nature has something to say on this question, and she is not by any means so stupid a pedagogue as many of our modern laboratory men seem to think. It is she who impresses upon us the fact of hunger; and hunger is a great recuperator, inasmuch as it not only compels us to eat, but to eat sufficient for satisfaction and the recovery of strength. Nature, too, at the close of each day, locks us fast in the recuperating chamber of sleep. In the earlier days of his life's experience the human being cannot help sleeping even if he would. At the close of the day, or near it, the infant, the child, and the unsophisticated man and woman quietly, happily, unconsciously pass into dreamless slumber. "The death of each day's life" opens upon a resurrection of pleasure, strength, and health under these natural circumstances.

Can anything be more entirely rational, scientific, and modern than this lesson in daily recuperation which nature teaches us and enforces upon us? But of what use is such elementary teaching to me? says the man who wants to work three or four hours longer every day than he ought to work, who insists upon undertaking the burdens and anxieties that half a-dozen people ought to bear, and who generally measures his work, not by his powers, but by his ambitions and competitive instincts. Such a man demands a method of recuperation which will enable him to carry out all the schemes which his impulses prompt him to enter upon, without any regard whatever to the measure of his capacity. The best answer to a demand of this kind is the shortest. The thing cannot be done. There are no methods of recuperation which will enable a man of only six-horse business power to do the work of a man of twelve-horse power.

But, it may be asked, are we then to be left entirely to the light of nature in the matter of recuperation? Has medicine nothing whatever to say upon the subject? Medicine has everything to say; but what she says first of all is this: That her principal business is to interpret nature to the non-medical world, and to reconcile impulsive, ignorant, and wilful men to the unyielding, but quite reasonable demands of nature. Medicine insists upon daily recuperation by means of food, rest, and sleep. Where the necessity arises, physic must be called in as an aid to nature, to promote appetite or digestion, sleep or rest. It will sometimes happen that the imperative demands of business will encroach upon the means of recuperation for a longer or shorter period. What is then to be done? One thing, and one thing only. The debt thus contracted, be it great or small, must be paid back in full, and sometimes even with interest. Whenever a man has been compelled to borrow from sleep or from recreation in order to satisfy the demands of business, he must, as soon as possible, pay back from business that which he borrowed from recreation and sleep. In no other way can physiological accounts be balanced and bankruptcy avoided. Medicine, as we have said, is like religion, a reconciler. Happy is the man who has submitted himself to her reasonable reconciliation.

